# Responses of plant leaf economic and hydraulic traits mediate the effects of early- and late-season drought on grassland productivity

**DOI:** 10.1093/aobpla/plz023

**Published:** 2019-04-04

**Authors:** Amarante Vitra, Claire Deléglise, Marco Meisser, Anita C Risch, Constant Signarbieux, Lia Lamacque, Sylvain Delzon, Alexandre Buttler, Pierre Mariotte

**Affiliations:** 1 Ecole Polytechnique Fédérale de Lausanne (EPFL), School of Architecture, Civil and Environmental Engineering (ENAC), Laboratory of Ecological Systems (ECOS), Switzerland; 2 Swiss Federal Institute for Forest, Snow and Landscape Research (WSL), Site Lausanne, Lausanne, Switzerland; 3 Agroscope, Grazing Systems Group, Route de Duillier, Nyon, Switzerland; 4 Université Grenoble Alpes, Irstea, LESSEM, Grenoble, France; 5 Swiss Federal Institute for Forest, Snow and Landscape Research (WSL), Community Ecology, Zuercherstrasse, Birmensdorf, Switzerland; 6 Université Clermont Auvergne, INRA, PIAF, Clermont-Ferrand, France; 7 ITEIPMAI, Domaine de la Vesc, Montboucher-sur-Jabron, France; 8 UMR BIOGECO, INRA–UB, University of Bordeaux 1, Bat, Talence, France; 9 Laboratoire de Chrono-Environnement, UMR CNRS 6249, UFR des Sciences et Techniques, Université de Franche-Comté, Besançon, France

**Keywords:** Drought timing, grassland productivity, plant functional traits, plant hydraulic status, precipitation manipulation, rainout shelter, vegetation dynamics, water limitation

## Abstract

Drought can occur at different times during the grassland growing season, likely having contrasting effects on forage production when happening early or later in the season. However, knowledge about the interacting effects of the timing of drought and the development stage of the vegetation during the growing season is still scarce, thus limiting our ability to accurately predict forage quantity losses. To investigate plant community responses to drought seasonality (early- vs. late-season), we established a drought experiment in two permanent grasslands of the Swiss Jura Mountains that are used for forage production. We measured three plant functional traits, including two leaf traits related to plant economics (specific leaf area, SLA; leaf dry matter content, LDMC) and one hydraulic trait related to physiological function (predicted percentage loss of hydraulic conductance, PLCp), of the most abundant species, and plant above-ground biomass production. Plant species composition was also determined to calculate community-weighted mean (CWM) traits. First, we observed that CWM trait values strongly varied during the growing season. Second, we found that late-season drought had stronger effects on CWM trait values than early-season drought and that the plant hydraulic trait was the most variable functional trait. Using a structural equation model, we also showed that reduction in soil moisture had no direct impacts on above-ground biomass production. Instead, we observed that the drought-induced decrease in above-ground biomass production was mediated by a higher CWM PLCp (i.e. higher risk of hydraulic failure) and lower CWM SLA under drought. Change in CWM SLA in response to drought was the best predictor of community above-ground biomass production. Our findings reveal the importance of drought timing together with the plant trait responses to assess drought impacts on grassland biomass production and suggest that incorporating these factors into mechanistic models could considerably improve predictions of climate change impacts.

## Teaser

Plant hydraulic traits measured at the community scale have rarely been used to assess grassland responses to drought. Here, the use of percentage loss of conductivity (PLCp) as a plant community hydraulic trait allowed us to observe its interactions with more commonly used plant leaf economic traits (SLA, LDMC) and its direct effects on grassland productivity under drought. Using a structural equation model, we showed that reduction in soil moisture had no direct impacts on above-ground biomass production. Instead, we observed that the drought-induced decrease in above-ground biomass production was mediated by a higher PLCp and lower SLA under drought.

## Introduction

As water availability is a main driver of net primary production, extreme drought events that are forecasted to increase in intensity and frequency within the century (Easterling *et al.* 2000; IPCC 2013) could severely reduce ecosystem productivity (Ciais *et al.* 2005; Calanca *et al.* 2014). This can have important economic consequences, notably in grasslands, which are the core areas for forage production worldwide. However, temperate grasslands commonly used for forage production seem to vary in their sensitivity to drought (Knapp *et al.* 2001), likely due to the diversity of grassland botanical composition, management practices, soil properties and local climatic conditions (Smith 2011; Thébault *et al.* 2014). Previous studies showed that grasslands at low annual precipitation sites (Gilgen and Buchmann 2009) or under intensive management practices (Vogel *et al.* 2012; Zwicke *et al.* 2013; Deléglise *et al.* 2015) could be more sensitive to drought. By contrast, grassland communities with higher diversity (Kahmen *et al.* 2005), higher abundance of subordinate plant species (Mariotte *et al.* 2013; Mariotte *et al.* 2016) or that are more limited by other resources than water (Huxman *et al.* 2004; Knapp *et al.* 2015) might be better in resisting drought.

Drought duration, intensity and timing (Knapp *et al.* 2002; Bloor *et al.* 2010; Zwicke *et al.* 2013; Denton *et al.* 2017), as well as frequency of rainfall events (Heisler-White *et al.* 2009; Didiano *et al.* 2016), timing (Chou *et al.* 2008) and intervals between rainfall events (Fay *et al.* 2000) are likely very important factors influencing the response of grassland communities to precipitation changes. Much uncertainty remains on how the seasonal pattern of drought will evolve in the future and only very few studies assessed the impact of the timing of drought during the vegetation growing season in grasslands (Dietrich and Smith 2016; Denton *et al.* 2017). Using a mesocosm experiment, De Boeck *et al.* (2011) compared how different timing of drought (i.e. spring, summer, autumn) affected experimental plant communities and showed that drought-induced reductions in plant growth and biomass were smaller in spring than in autumn, but stronger in summer. The timing of drought can thus strongly influence how grassland communities respond to water scarcity but its effects remain poorly investigated in natural field conditions.

Plant functional traits have been shown to strongly vary along soil moisture gradients (Cornwell and Ackerly 2009; Bruelheide *et al.* 2018; Griffin-Nolan *et al.* 2018), and thus could be good indicators of the plant response to drought (Garnier *et al.* 2001). At the community level, the functional characteristics of the most abundant species are expected to be the main driver of ecosystems processes (i.e. mass-ratio hypothesis, Grime 1998). Therefore, determining community-weighted mean (CWM) traits can be a relevant tool to assess drought effects on grassland communities as they express both trait variability due to intraspecific variability and changes in species composition and abundances (Garnier *et al.* 2004; Violle *et al.* 2007). In temperate grasslands, previous studies showed that leaf traits related to plant economics strongly respond to drought, with specific leaf area (SLA) decreasing and leaf dry matter content (LDMC) increasing with increasing dryness (Buckland *et al.* 1997; Volaire 2008; Poorter *et al.* 2009; Jung *et al.* 2014; Deléglise *et al.* 2015; Wellstein *et al.* 2017). Changes in these plant functional leaf traits are integrative of the whole stress period and reflect structural changes in plant tissues with direct consequences for plant biomass production (Pontes *et al.* 2007; Griffin-Nolan *et al.* 2018).

Recently, Brodribb (2017) called for using more mechanistic functional traits to assess plant responses to environmental perturbations, since such traits can directly and immediately represent the physical mechanisms of the water movement and status experienced by the plants during drought. Therefore, plant hydraulic traits can be good indicators of immediate response to drought at the plant level and can directly reflect the mechanistic responses related to physiological functions. For example, minimum xylem water potential (PSI_min_), midday water potential (Ψ_midday_) or the water potential leading to 50 % loss of hydraulic conductance (*P*_50_) have long been used to characterize tree strategies in response to drought (Tyree *et al.* 1992; Cochard *et al.* 1996; Choat *et al.* 2012; Anderegg *et al.* 2016). Both Ψ_midday_ and *P*_50_ values can be used to determine the predicted percentage loss of hydraulic conductance (PLCp) in the field, an important plant hydraulic trait in resistance to water scarcity. However, plant hydraulic traits have been poorly assessed in herbaceous angiosperm species (Pérez-Ramos *et al.* 2013; Lens *et al.* 2016; Griffin-Nolan *et al.* 2018) despite such traits directly affecting plant growth and thus biomass production through impacts on carbon assimilation and cell expansion (Basra 1997; Chaves and Oliveira 2004).

Despite an extensive literature on drought impacts in various ecosystems, research on leaf economic and hydraulic traits’ responses to drought has developed, for the most part, independently, and linkages between both types of traits remain poorly understood. Few studies on trees that experienced prolonged periods of drought highlighted strong links between traits related to plant economics (i.e. SLA and LDMC) and hydraulic traits (e.g. Vinya *et al.* 2012; Gilbert and Medina 2016). For example, high LDMC might confer the ability to plants to withstand lower negative leaf water potential and contribute to maintaining physiological processes during drought (Kursar *et al.* 2009; Vinya *et al.* 2012). On the other hand, increased loss of conductivity (PLC), as a response to drought, can reduce SLA through a decrease in water transport to the leaf (Villagra *et al.* 2013). The effects of drought, and also timing of drought, on the links (i.e. positive or negative correlation) between these different plant traits can thus have important consequences for biomass production along the plant growing season **[see****Supporting Information—Fig. S1****]**. However, such linkages have never been investigated in grasslands, and more particularly in mowed grasslands that are largely used for forage production and cattle feeding.

With a few exceptions (Coleman *et al.* 1994), there is a general lack of knowledge about the seasonal and inter-annual variability in plant functional traits (Griffin-Nolan *et al.* 2018). However, temporal trait variability (i.e. whether trait value is high or low) is likely to impact plant traits’ responses to extreme climatic events such as drought during the vegetation growing season. Therefore, in this study, we first assessed the variability in CWM leaf (SLA, LDMC) and hydraulic (PLCp) plant traits at two permanent grassland sites with similar mowing practice but contrasted soil characteristics during 2 years. We expected strong fluctuations in plant trait values due to seasonal and annual climatic conditions across sites. Second, we tested how the timing of a drought during the growing season impacts CWM traits depending on the trait values at the time of drought. At both sites, the amount of precipitations was manipulated by using rainout shelters, simulating an early- or a late-season drought. We hypothesized that drought occurring early in the season, at peak of biomass production, would more strongly affect plant traits, as plant tissues are more active at this period compared to a drought happening after the peak of biomass production (Cornwell and Ackerly 2009). Finally, we aimed at determining which of the plant functional traits would be most responsive to water scarcity and better explain changes in grassland biomass production under drought.

## Materials and Methods

### Study sites

**Figure 1.**
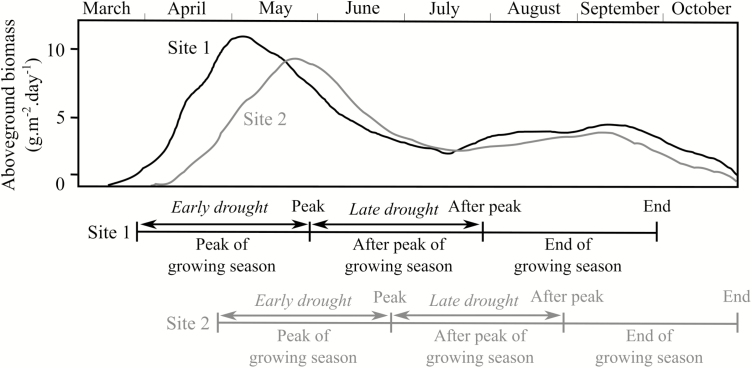
Scheme of the seasonal dynamic of grassland biomass production for the two sites (unpublished data available from Agroscope institute). The different periods of plant growth along the season (each lasting for 2 months) are represented below the graph (Peak of growing season, After peak of growing season and End of growing season) with their respective sampling times (Peak, After Peak and End) for plant trait measurements and above-ground biomass harvest. The ‘peak of growing season’ period has been centred on the peak of the vegetation growth curve and determined the beginning of the experiment at each site. The growth curve, periods and sampling times are drawn in black for Site 1 and grey for Site 2. Drought was applied either during the peak of growing season (i.e. Early drought) or after the peak of growing season (i.e. Late drought). Mowing occurred at the sampling times (Peak, After Peak and End) to simulate common management practices at both sites.

The experiment was conducted from spring 2015 to fall 2016 at two permanent grassland sites: Site 1 at Chéserex (46°24′N, 6°10′E) and Site 2 at Saint-George (46°30′N, 6°15′E). The sites are located in the Swiss Jura Mountains at 540 and 940 m above sea level, respectively. Climate at the two sites is suboceanic with mean annual precipitation of 1050 and 1290 mm and mean annual temperatures of 10.4 °C and 7.6 °C (averaged 1981–2010, MeteoSwiss) at Sites 1 and 2, respectively. Mean precipitation (averaged 1981–2010, MeteoSwiss, ±95 % confidence intervals) during the period of our precipitation manipulation experiment (i.e. plant growing season, 6 months, see Fig. 1) was 441 ± 36 mm at Site 1 and 682 ± 46 mm at Site 2, and mean daily average temperatures were 16 ± 0.3 °C at Site 1 and 13.5 ± 0.3 °C at Site 2 (see also Buttler *et al.* 2019). Despite receiving different amount of precipitation during the plant growing season, both sites experience similar rainfall frequency with about 11 rainy days per months with precipitation equally spread over the season (see Buttler *et al.* 2019). Soils at Sites 1 and 2 were both classified as cambisols [World Reference Base for Soil Resources—IUSS Working Group WRB (2006)] but are quite different in depth, organic matter (OM) content, N and P availability. Site 1 has a deeper soil (90 cm) and is characterized by 19.8 % clay, 41.2 % silt and 38.9 % sand, a pH of 5.8 and 4.7 % OM. Site 2 has a rather shallow soil (45 cm depth) and is characterized by 36.3 % clay, 41.5 % silt and 24.2 % sand, a pH of 7.5 and 8.5 % OM.

The botanical composition of the two sites was similar and dominated by perennial grasses (*Lolium perenne*, *Dactylis glomerata*, *Poa pratensis*, *Phleum pratense*) and forbs (*Trifolium repens* and *Taraxacum officinale*) which all accounted for at least 80 % of the plant biomass. At both sites the ground is regularly covered by snow or frozen from about November to March; thus, plant above-ground parts (stem and leaves) are senescent during winter and the vegetation growing season starts in April and ends in October. Following the common practice of the region, the two sites were managed with mowing every 2 months during the plant growing season and fertilized with commercial organic manure (5.2 % organic nitrogen and 4.4 % phosphate) added in split applications, half amount in spring and half amount in autumn. Both sites are highly productive with an average annual biomass production of 900–1200 g dry matter per m^2^.

### Experimental drought manipulation

An identical precipitation manipulation experiment with three drought treatments was carried out at the two grassland sites: Control (no drought), early-season drought event (hereafter called ‘Early’) and late-season drought event (hereafter called ‘Late’). At the beginning of the experiment, five replicated rainout shelters (length: 12 m; width: 6.4 m, height: 3 m, Filclair, Numeris 6.40, Venelles, France) covered with a transparent plastic film (180 μm, transparent M42, Filclair, Venelles, France) were established at each site. Three plots of 4 m × 0.9 m (separated by 80 cm) corresponding to the three drought treatments (i.e. Control, Early and Late) were randomly installed under each rainout shelter.

Control plots were watered according to the average precipitation of the last 30 years received at each site (i.e. 441 mm at Site 1 and 682 mm at Site 2 over the 6 months of the experiment). Drought plots simulated rainfall conditions according to the intermediate scenario of climatic models in our study region (CH2011 2011). The Early drought treatment consisted in a reduction of precipitation that occurred during 2 months centred on the peak of plant growing season (Fig. 1). For the Late drought treatment, the reduction in precipitation occurred for 2 months after the peak of plant growing season (Fig. 1). During the respective drought event, plots only received 30 % of the water added in the control plots. Outside the Early or Late drought-induced periods, plots received the same amount of water as the control plots until the end of the growing season (hereafter called ‘End’), which corresponded to the 2 months after the end of the late-season drought (Fig. 1). Detailed precipitation data during (2 months) and after the peak (2 months) of plant growing season, as well as until the end of the growing season (2 months), are available in Buttler *et al.* (2019). Watering was done manually, every 2–3 days in control plots to simulate the rainfall frequency of the region (i.e. about 11 rainy days per months during the plant growing season), and every 4–5 days in drought plots to simulate 50 % decrease in rainfall frequency, which is expected to occur simultaneously with precipitation reduction (CH2011 2011). Experimental methods were the same in both years and rainout shelters were in place from 31 March to 15 September at Site 1 and from 23 April to 7 October at Site 2 in 2015 and from 9 April to 24 September at Site 1 and from 21 April to 6 October at Site 2 in 2016. The sites received ambient precipitation for the rest of the year. Soil moisture was measured one time a week with a time domain reflectometer (FieldScout TDR 100 Soil Moisture Meter) for the top 15 cm of the soil. Three randomly located moisture measurements per plot were averaged. The mean air temperature in 2015 was 13.9 °C and 21.2 °C for Site 1 during and after the peak of growing season, respectively, and 13.0 °C and 19.2 °C for Site 2. In 2016, mean air temperature during and after the peak of growing season was 10.5 °C and 17.9 °C, respectively, for Site 1 and 9.9 °C and 16.7 °C, respectively, for Site 2.

### Plant biomass harvest and community composition

During our experiment, all plots were managed according to the forage conservation regime (hay making) typical for the region; plots were mowed to a height of 5 cm every 2 months. Mowing occurred three times per year, the first at the end of the peak of growing season (i.e. Peak, also corresponding to the end of the Early drought), the second 2 months after the end of growing season (i.e. After peak, also corresponding to the end of the Late drought) and the third at the end of the growing season (i.e. End, see Fig. 1). It is important to note that due to the managing practice in these grasslands, the measurements during the peak of growing season were performed on the first growth each year, whereas the ones made after the peak of growing season concerned the regrowth cycle after mowing. In each plot, above-ground biomass was collected from a 65 × 400 cm subplot at the same time as mowing (i.e. at the three sampling times: Peak, After Peak, End). These samples were dried at 60 °C for 72 h, then at 105 °C for 3 h and weighed. Plant above-ground biomass was expressed in g m^−2^.

Botanical surveys were performed few days before the biomass harvest using the *Daget–Poissonet* method (Daget and Poissonet 1971) with 80 points per plot, evenly distributed every 20 cm on four lines of 400 cm spaced 20 cm apart. Altogether, the four lines covered the entire plot, leaving 10–15 cm between lines and the border of the plot to avoid edge effects. At each point of interception, we recorded all plant species in contact with the edge of a 1 mm dagger (presence/absence) without taking into consideration the number of contacts. Relative species cover was determined by dividing the number of contacts per species in each plot by the total number of contacts.

### Plant CWM functional traits

At both sites, we selected the most abundant plant species accounting for at least 80 % of the biomass at the beginning of the experiment (spring 2015): *D. glomerata*, *L. perenne*, *P. pratensis* and *T. repens* at both sites, plus *P. pratense* and *T. officinale* at Site 1. One day before mowing, we measured three plant traits (one hydraulic trait and two leaf economic traits) for each selected species at both sites, for the three drought treatments (Control, Early, Late) at the three sampling times (Peak, After peak, End, see Fig. 1) in both years (2015, 2016).

The predicted percentage loss of hydraulic conductance (PLCp, %), used as the plant hydraulic trait, was derived from the Ψ_midday_ and vulnerability curves (VCs) for the studied six species. Prior to the field experiment, we first determined xylem resistance to embolism for each species (Lens *et al.* 2016). For this purpose, we collected between 20 and 30 flowering stems of different individuals for each plant species at both sites in 2015, wrapped them into wet papers and immediately sent them to the Caviplace platform (Delzon Lab, UMR Biogeco, University of Bordeaux, France) where it arrived within 48 h. Samples were not flushed with water in order to avoid possible effects of air-seeding fatigue due to a stretching or degradation of the pit membranes during previous embolism events (Li *et al.* 2016) but all samples were well hydrated when measuring xylem resistance to embolism. Different techniques exist to measure xylem hydraulic conductivity (Melcher *et al.* 2012). For example, x-ray microtomography observation (Cochard *et al.* 2015) and optical vulnerability technique (Brodribb 2017) allow direct and real-time visualization of embolism through the vascular system but are difficult to access or time-consuming. By contrast, *in situ* flow centrifuge technique is an indirect method based on the assessment of the relative decrease in xylem transport efficiency caused by the presence of air in the conduits. However, this method is quicker than any other techniques (Cochard *et al.* 2005, 2013) and this is the method we used to determine VCs for each species. To increase the water flow, between 4 and 8 stems (depending on species) were grouped in a bunch and spun at the same time (see Lens *et al.* 2016). From these curves, the *P*_50_ was determined, which corresponds to the sap tension (MPa) inducing 50 % loss of hydraulic conductance (Cochard *et al.* 2013; see Lens *et al.* 2016). As VCs and *P*_50_ are considered intrinsic traits at the species level (Lamy *et al.* 2014, but see Anderegg 2015), these measurements were only done in 2015, and indeed similar curves and *P*_50_ were observed for the same species at both sites. Second, we measured midday leaf water potential (Ψ_midday_) on the same species in our field experiment. Ψ_midday_ was obtained by averaging measures performed on the first fully expanded leaf from flowering stalk of three individuals per species. The measurements were conducted between 11 a.m. and 3 p.m. on sunny days using a Scholander pressure chamber (SKPM, Skye Instruments Ltd, Powys, UK) for each species under each drought treatment and at the different sampling times. The PLCp values were then estimated as follow:

$$\begin{array}{l} PLCp=\frac{100}{1+e^{\left(\frac{Slope}{25}*({\;\Psi \;}_{midday}-P_{50})\right)}} \end{array}$$

with Slope being the slope of the VC of a specific species, Ψ_midday_ being the leaf water potential (MPa) experienced by the species in the field and *P*_50_ the water potential inducing 50 % loss of hydraulic conductance (MPa) for the species (see Urli *et al.* 2015).

Leaf dry matter content was measured according to the protocol of Cornelissen *et al.* (2003). The youngest fully mature leaf on five mature individuals was sampled for each species. Leaves were kept in plastic bags with few drops of deionized water for at least 24 h at 4 °C to allow plant tissues to rehydrate (Garnier *et al.* 2001). We then weighed the samples to record their water-saturated fresh weight (FW). Afterwards the samples were dried at 60 °C and their dry weight (DW) was recorded after 72 h. Leaf dry matter content was then calculated as DW divided by FW.

$$\begin{array}{l} LDMC\;(mg\;g^{-1})=DW\;(mg)/FW\;(g) \end{array}$$

Specific leaf area was measured according to (Cornelissen *et al.* 2003) using the leaves collected for LDMC described above. We determined the leaf surface of all the plant samples by using a planimeter (LI-COR, LI 3000C Portable Area Meter), allowing us to calculate SLA as the one-sided area of a fresh leaf divided by its DW.

$$\begin{array}{l} SLA\;(cm^{2}\;g^{-1})=leaf\;surface\;(cm^{2})/DW\;(g) \end{array}$$

In a final step, we calculated the CWM traits of PLCp, LDMC and SLA (Garnier *et al.* 2004) as the sum of the average value of the traits (per species) multiplied by the relative abundances of the species (*p*_*i*_, %) divided by the sum of the relative abundances of the *n* species:

$$\begin{array}{l} CWM=\frac{\;\;\;\overset{n}{\underset{i=1}{\sum }}p_{i}*trait_{i}}{\overset{}{\sum }p_{i}} \end{array}$$

### Plant community responses to drought

To highlight the effects of drought on plant traits and biomass and to compare the amplitude of the effects at the different periods of the growing season and in both years, we determined the response ratios (RRs) as follow:

$$\begin{array}{l} RR=\frac{CWM_{D}-CWM_{C}}{CWM_{C}\;} \end{array}$$

with $CWM_{D}$ corresponding to the CWM traits at the end of the drought treatment (Early or Late) and $CWM_{C}$ corresponding to the CWM traits in the respective control plot at the same date. A RR > 0 means that the CWM trait increased in the drought compared to the control plots. By contrast, a RR < 0 means that the CWM trait decreased in the drought compared to the control plots. Response ratio corresponds to percentage of change in drought compared to control plots (e.g. RR = −0.20 means 20 % decrease in trait value under drought). Response ratios were calculated for CWM PLCp, CWM LDMC, CWM SLA and for the plant biomass production by using the above-ground biomass data in drought and control plots.

### Statistical analysis

All analyses were carried out with R version 3.4.0 (R Development Core Team 2017) and data were analysed separately for both sites. Seasonal (Season: Peak, After peak, End) and inter-annual (Year: 2015, 2016) variability effects on CWM traits in control plots were tested using linear mixed-effect model (packaged ‘nlme’) specifying ‘block’ as random factor. Data of PLCp were log transformed to comply with the assumptions of normality and homoscedasticity.

We performed *t*-tests on RRs for all CWM traits and above-ground biomass at each sampling time and year to ensure responses to drought were significantly different from zero. To test for the effect of the timing of drought (Early vs. Late) on the RR of plant traits and above-ground biomass to drought, we used only the data for the early-season drought at the peak of growing season (Early at Peak) and the late-season drought after the peak of growing season (Late at After peak) for both years, which corresponds to the drought effects at the end of the respective drought treatments. Effects of drought timing (DT: Early, Late), years (Year: 2015, 2016) and their interactions on the RR of the CWM and biomass production were tested using a linear mixed-effect model specifying ‘block’ as random factors.

We ran linear regressions between the RR of above-ground biomass production, the RR of CWM PLCp, CWM SLA and CWM LDMC to drought to test for the links between the response of plant hydraulic traits, plant functional traits and above-ground biomass production to drought. Regressions include data for both years (2015, 2016) and the three sampling dates (Peak, After peak, End) and were performed separately for Sites 1 and 2. Statistical significance of linear regressions was obtained from linear mixed-effect model specifying ‘Season’ nested into ‘Year’ nested into ‘Block’ as random factors, thus accounting for repeated measures sampling. Coefficient of determination (*R*^2^) for linear mixed-effect models was determined using the function ‘r2beta’ of the package ‘r2glmm’ (Jaeger *et al.* 2017).

Complex interactions between the relative effects of drought on soil moisture and the response of plant functional leaf and hydraulic community traits and plant community biomass to drought (i.e. RR for all parameters) were analysed through structural equation modelling (Grace *et al.* 2014). We used a path analysis approach, a particular case of structural equation modeling involving only quantified variables, to test for the effect of soil moisture reduction resulting from our drought manipulation on the linkages between plant leaf and hydraulic traits and plant biomass production. Using *a priori* knowledge based on the literature introduced above, we built a network of causal relationships among all measured variables **[see****Supporting Information—Fig. S1****]**. Models were fit using maximum likelihood estimation with robust SE and Satorra–Bentler scaled test statistic with the ‘lavaan’ package (Rosseel 2012). Then, the successive full model was simplified by stepwise exclusion of variables with non-significant weights and non-significant covariance, until a minimal adequate model showing specific linkages remained, estimated by the lowest Akaike information criterion. *Z*-statistic was used to determine the significance of each pathway. Final model fits were assessed with the chi-square test (*P* > 0.05), the root mean square error of approximation index (RMSEA < 0.05), the low standardized root mean square residual index (SRMR < 0.05) and high comparative fit index (Grace *et al.* 2010).

## Results

### Seasonal and inter-annual variability in CWM plant traits and biomass production

**Figure 2.**
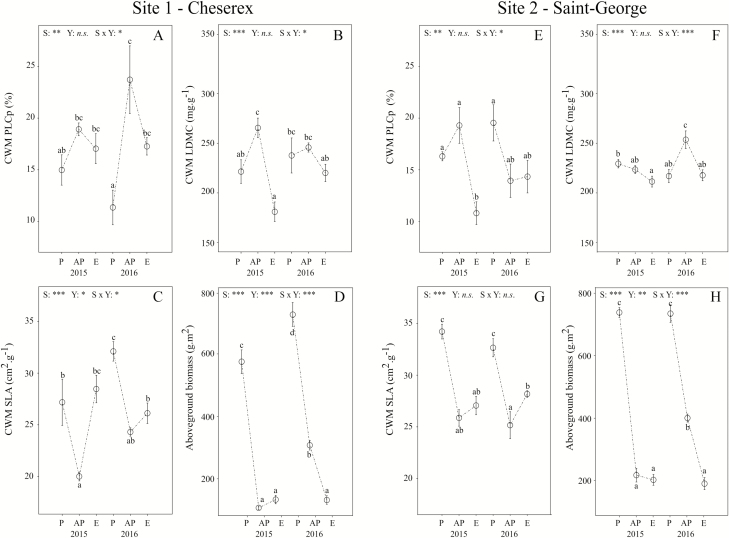
Seasonal and inter-annual variability in CWM plant traits [PLCp: predicted percentage loss of hydraulic conductance (A, E), LDMC: leaf dry matter content (B, F), SLA: specific leaf area (C, G)] and biomass production (D, H) along the plant growing season (P: Peak, AP: After peak, E: End) at both sites and for both years. Within each year Peak and After peak, as well as After peak and End, are separated by 2 months. Statistical results are displayed for the effects of the season (S: Peak, After peak, End), the year (Y: 2015 vs. 2016) and their interaction (S × Y) and significant effects are indicated in each graph (*P < 0.05, **P < 0.01, ***P < 0.001).

All the CWM traits, except CWM SLA at Site 2, differed across the seasons and between the 2 years (i.e. significant Season × Year interaction, Fig. 2) in the control plots (i.e. no drought simulation). At Site 1, CWM PLCp was higher after the peak of growing season in both years, particularly in the second year where it reached 23.71 % (Fig. 2A), and much lower at the end of the growing season. Opposite effects were found for CWM SLA (Fig. 2C), which decreased after the peak of growing season and increased at the end of the vegetation season. CWM LDMC increased after the peak of growing season during the first year and decreased in both years at the end of the vegetation season (Fig. 2B).

At Site 2, CWM PLCp reached its maximum after the peak of growing season during the first year and at the peak of growing season during the second year (Fig. 2E). CWM LDMC increased after the peak of growing season during the second year only but decreased on both years at the end of the vegetation season (Fig. 2F). CWM SLA decreased after the peak of growing season in both years and increased at the end of the vegetation season in the second year (Fig. 2G). Additional analysis at species level at both sites reveals that seasonal and inter-annual variability in CWM traits was due to variations in both relative abundance and plant trait values of the most abundant species within the plant community **[see****Supporting Information—Table S1****]**.

At both sites, above-ground plant biomass strongly decreased after the peak of growing season (Fig. 2C and H) but less so during the second year of the experiment (i.e. 2 months after the peak of growing season) as indicated by the significant Season × Year interaction (Sites 1 and 2, *P* < 0.001).

### Effect of precipitation manipulation on soil moisture

The experimental manipulation of precipitation resulted in a decrease of soil moisture in the plots under drought by comparison to the control plots. This decrease in soil moisture ranged from −34 % at the end of the late drought treatment in 2016 at Site 1 to −63 % at the end of the early drought treatment in 2015 at Site 2 **[see****Supporting Information—Table S2****]**. During the 2-month period that followed both the early and late drought treatments, soil moisture increased due to rewetting, but was still always lower than in the control plots.

### Drought impacts on CWM plant trait values and biomass production

Drought effects on CWM traits assessed by RRs depended on the trait considered, the timing of the drought and the site (Fig. 2). It is important to note that, for both sites, drought had no effects on the relative abundance of species **[see****Supporting Information—Figs S2****and****S3****]**. Thus changes in CWM traits as a response to drought were mainly influenced by changes in plant trait values.

**Figure 3.**
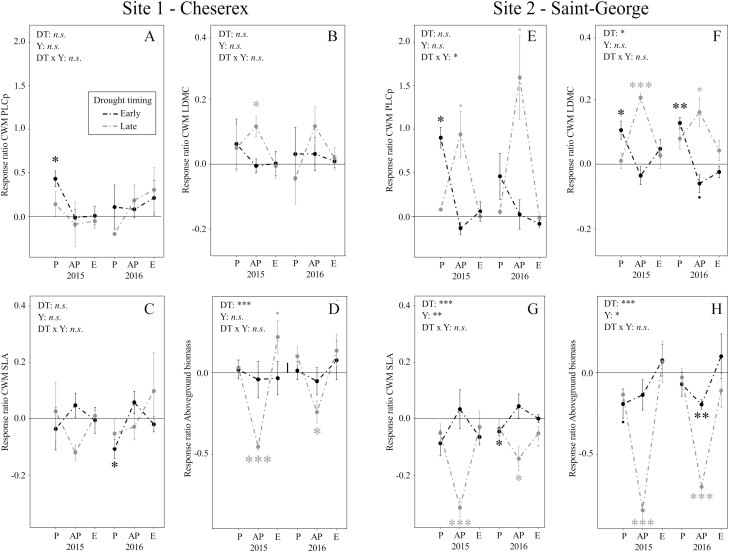
Response ratio of CWM plant traits [PLCp: predicted percentage loss of hydraulic conductance (A, E), LDMC: leaf dry matter content (B, F), SLA: specific leaf area (C, G)] and biomass production (D, H) to early- (Early; black circle) and late-season drought simulation (Late; grey circle) along the plant growing season (P: Peak, AP: After peak, E: End) at both sites and for both years. Within each year Peak and After peak, as well as After peak and End, are separated by 2 months. Statistical results are displayed for the effects of the timing of drought (DT: Early vs. Late), the year (Y: 2015 vs. 2016) and their interaction (DT × Y) and indicated for RRs significantly different from zero. Significant effects are indicated in each graph (*P* < 0.10, *P < 0.05, **P < 0.01, ***P < 0.001).

Overall, CWM PLCp (Fig. 3A and E) and CWM LDMC (Fig. 3B and F) increased with drought, while CWM SLA (Fig. 3C and G) and above-ground biomass production (Fig. 3D and H) decreased with drought. At Site 1, CWM PLCp significantly increased during the early-season drought in 2015 (Fig. 3A), CWM LDMC increased during the late-season drought in 2015 (Fig. 3B) and CWM SLA decreased during the early-season drought in 2016 (Fig. 3C). Community above-ground biomass only decreased during the late drought at Site 1 during both years (Fig. 3D).

Overall, the CWM traits (LDMC, PLCp or SLA) were more affected by drought at Site 2 compared to Site 1 and the timing of the drought only yielded different effects on CWM traits (LDMC, PLCp or SLA) at Site 2 (Fig. 3E–G). At Site 2, CWM PLCp was higher during the early-season drought in 2015 and marginally higher during the late-season drought in both years (Fig. 3E). CWM LDMC significantly increased (Fig. 3F) and CWM SLA decreased (Fig. 3G) during the early- and late-season drought regardless of the year of sampling. Furthermore, CWM PLCp was more strongly affected during the late- than the early-season drought in the second year of the experiment (Fig. 3E), as highlighted by the significant Timing of Drought × Year interaction (*P* < 0.05). We also found a greater increase in CWM LDMC (Fig. 3F; Timing of Drought, *P* < 0.05) and greater decrease in CWM SLA (Fig. 3G; Timing of Drought, *P* < 0.01) during the late- than during the early-season drought in both years. Above-ground plant biomass decreased for both drought simulations at Site 2 (Fig. 3H) but more strongly during the late- than the early-season drought for both years.

### Relationships between plant traits and plant biomass

**Figure 4.**
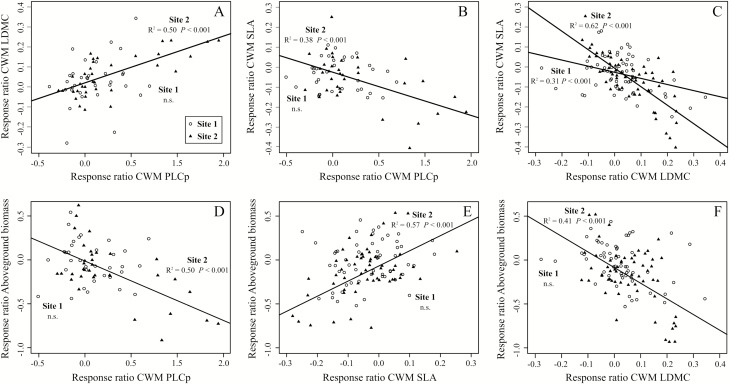
Linear relationships between RRs to drought for the different CWMs of traits and above-ground biomass (A) CWM LDMC and CWM PLCp, (B) CWM SLA and CWM PLCp, (C) CWM SLA and CWM LDMC, (D) above-ground biomass production and CWM PLCp, (E) above-ground biomass production and CWM SLA, (F) above-ground biomass production and CWM LDMC. Black circles and black triangles correspond to the values of Site 1 (Chéserex) and Site 2 (Saint-Georges), respectively. Regressions include data for both years 2015 and 2016 and the three sampling dates (Peak, After peak, End). Statistical significance of regressions (*P-*value) and *R*^2^ were assessed using linear mixed-effect models accounting for repeated measures.

The responses to drought of CWM LDMC (Fig. 4A; *P* < 0.001, *R*^2^ = 0.50) and CWM SLA (Fig. 4B; *P <* 0.001, *R*^2^ = 0.38) were significantly correlated to the response of the CWM PLCp at Site 2 (positively with LDMC and negatively with SLA), but were not significantly correlated for Site 1. The responses of the CWM SLA and CWM LDMC to drought were significantly and negatively correlated at both Site 1 (*P <* 0.001, *R*^2^ = 0.31) and Site 2 (*P <* 0.001, *R*^2^ = 0.62) (Fig. 4C). The RR of the above-ground biomass to drought was significantly negatively correlated to RR of CWM PLCp (Fig. 4D; *P* < 0.001, *R*^2^ = 0.50) and CWM LDMC (Fig. 4F; *P* < 0.001, *R*^2^ = 0.41), and positively correlated to the CWM SLA (Fig. 4E; *P* < 0.001, *R*^2^ = 0.57) at Site 2. These correlations were not significant at Site 1.

**Figure 5.**
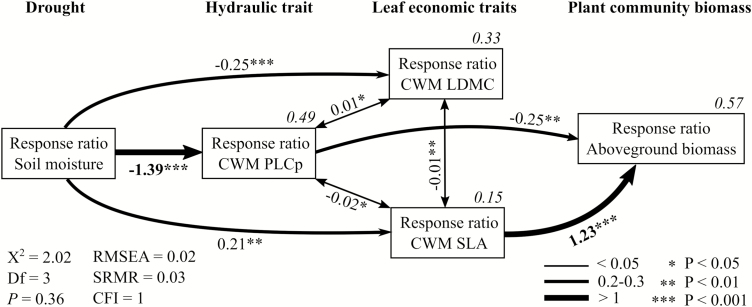
Minimal adequate structural equation model for the effects of soil moisture RRs on the linkages between CWM hydraulic trait RR (PLCp: percentage loss of hydraulic conductance), leaf economic trait RRs (SLA: specific leaf area; LDMC: leaf dry matter content) and above-ground biomass RRs to drought at Site 2 (Saint-George). The model highlights indirect effects of soil moisture reduction on above-ground biomass through changes in PLCp and SLA. Arrows shows significant relationships (pathways) between variables and numbers next to arrows show standardized parameter estimates (i.e. standardized regression weights). Square multiple correlations (*R*^2^ values) for the predicted/dependent factor are given on the box of the dependent variable.

Overall, our results show that relationships between the response of leaf and hydraulic traits and above-ground biomass were only expressed at Site 2 (Fig. 4), which was the site more strongly affected by our drought treatments. Structural equation modelling could therefore only be conducted for this site. The fitting parameters of the minimal adequate path analysis model (Fig. 5) indicate a good model fit (i.e. *X*^2^ = 2.02, *P* = 0.36, RMSEA = 0.02, SRMR = 0.03 and CFI = 1). The decrease in soil moisture (i.e. negative RRs of soil moisture) was strongly negatively correlated to the response of CWM PLCp (path = −1.39), thus highlighting the increase of PLCp with drought. The responses of CWM LDMC and CWM SLA to drought were negatively correlated and both directly affected by the decrease in soil moisture. The response of plant above-ground biomass was not directly related to the decreased soil moisture, but indirectly through the increase in CWM PLCp and the decrease in CWM SLA. The response of CWM SLA to drought (i.e. decreasing with decreasing soil moisture) was the strongest predictor (path = 1.23) of the response of plant above-ground biomass to drought (i.e. decreasing with decreasing soil moisture), followed by the response of CWM PLCp (path = −0.25), while the response of CWM LDMC had no direct influence.

## Discussion

Our study quantified variation in plant functional leaf and hydraulic traits in grassland communities over the course of two growing seasons. We determined how these traits were affected by experimental drought treatments applied at different times during the growing season (Early- vs. Late-season drought), and how the traits related to biomass production at the community level. We observed that the plant functional community traits (CWM) fluctuated under control conditions over the growing season, between years and also between the two experimental sites, despite the most abundant species being similar among sites. For example, a general trend occurring across sites is the lower CWM SLA and the higher CWM PLCp and CWM LDMC after the peak of growing season, which coincide with the longer and warmer summer days (June to August). Overall, combinations of seasonal and local climatic conditions, as well as management practices, likely explain the plant trait variability observed along the growing season and between sites. While there is extensive literature on plant traits (e.g. Garnier *et al.* 2004; Violle *et al.* 2007; Jung *et al.* 2014; Deléglise *et al.* 2015; Wellstein *et al.* 2017), such temporal and spatial variability over the growing season is less commonly documented. Therefore, our results highlight the need to account for sampling time in plant trait research, especially when comparing plant community traits between sites or years.

Our observed changes in CWM traits in response to drought were mainly related to changes in plant traits rather than changes in species abundance **[see****Supporting Information—Figs S2****and****S3****]**. Such results reflect plant species plasticity in response to our 2-year drought simulation (Lloret *et al.* 2012; Jung *et al.* 2014; Deléglise *et al.* 2015), rather than species turnover and community composition change, which would occur over longer drought perturbations (Smith *et al.* 2009). Interestingly, simulated drought events had stronger effects on plant traits and biomass production at Site 2, despite similar reductions in soil moisture at both sites. These differences could be explained by the difference in soil depth, 90 cm for Site 1 against 45 cm for Site 2, potentially allowing plants’ roots to grow deeper and reach water in deeper soil layers at Site 1. At Site 2, where the effects of drought were stronger, we found significant correlations between all the RRs of the CWM traits that we measured, as well as between the CWM traits and above-ground biomass production. We observed that LDMC increased and SLA decreased under drought as also shown in other studies (Jung *et al.* 2014; Deléglise *et al.* 2015). The strong correlation between these two plant functional traits relate to plant strategy as plants tend to invest either in leaf surface, i.e. higher SLA or leaf thickness, i.e. higher LDMC. Slower and thicker leaf growth is common in response to drought (Chaves *et al.* 2003; Gazanchian *et al.* 2007), as leaf development is hindered by water availability (Baker *et al.* 1985; Tardieu *et al.* 1999), mainly through the reduction in leaf cell expansion and cell division in meristems. In our study, we also observed that PLCp was positively correlated with leaf thickness (low SLA and high LDMC). Consequently, higher leaf thickness and increase in PLCp show that by limiting water transport through the xylem tissue (i.e. reduction of hydraulic conductance, PLCp), plant leaf tissue density is increasing as the leaf growth is limited. This increase in leaf density might allow plants to better resist drought due to higher water use efficiency, limited water loss (Wright *et al.* 2001; Xu and Zhou 2005; Aguirrezabal *et al.* 2006; Monclus *et al.* 2006) and longer leaf life (Poorter *et al.* 2009). It also enables leaves to maintain cell turgor (Nunes *et al.* 1989; Markesteijn *et al.* 2011).

Overall, our results revealed that the intensity of change in plant hydraulic (PLCp) and leaf (SLA and LDMC) community traits, as well as in above-ground biomass, was higher when a drought occurred after the peak biomass of production (when trait values are already at their lowest for CWM SLA and highest for CWM LDMC and CWM PLCp, see above). These results contrasted our initial hypothesis, but are consistent with the study of De Boeck *et al.* (2011), in which the authors found a stronger impact of drought in summer than in spring. These authors also showed that heat waves in summer were indirectly increasing the negative effect of drought on biomass production decrease. At both of our study sites, air temperatures were higher, and induced higher vapour-pressure deficit (VPD), during the late-season drought compared to the early-season drought (VPD = 0.78 vs. 0.47 KPa at Site 1 and VPD = 0.59 vs. 0.27 KPa at Site 2; see Buttler *et al*. 2019). Vapour-pressure deficit is a measure of the atmospheric demand for water, and similarly to soil moisture, directly influences vegetation water use and productivity (Novick *et al*. 2016; Konings *et al*. 2017). Indeed, to avoid excessive water loss when VPD is high, plants close their stomata, which also reduces carbon uptake. Therefore, higher VPD during late-season drought at both sites likely explains the stronger decrease in above-ground biomass compared to early-season drought.

The structural equation model helped to gain a better understanding of the relationships between the plant leaf and hydraulic community traits and above-ground biomass production in response to drought and showed that these relationships are much more complex than those initially suspected. Interestingly, reduction in soil moisture had no direct impacts on above-ground biomass production. Instead, we observed that the decrease in above-ground biomass production was partially due to a higher PLCp and lower SLA. As hypothesized by Pérez-Ramos *et al*. (2013), plants subject to drought can shed their leaves to lower the transpiring surface, which in turn could explain the direct effect of PLCp on above-ground biomass. Moreover, when PLCp is higher, plants are at higher risk of hydraulic failure, reducing the flow of water from roots to shoots. Less water being transported to the leaves can result in decreased biomass production. Change in SLA, one of the plant functional leaf traits, in response to drought was the best predictor of the response of above-ground biomass production. This is not surprising as most of the increase in above-ground biomass is due to an increase in leaf mass (Weraduwage *et al*. 2015).

## Conclusions

Our study showed a strong temporal (season, years) as well as spatial (sites) variability in plant community traits, due to natural fluctuation in species abundance and traits over time. These results show how important it is to consider spatio-temporal variability of community plant traits in future plant trait studies. We also demonstrated that in addition to natural spatio-temporal variability, a limitation in soil water availability impacted plant communities differently depending on when the drought occurred during the growing season. To our knowledge, plant hydraulic traits measured at the community scale have never been used to assess plant community response to drought in grasslands (see review by Griffin-Nolan *et al*. 2018). Here, the use of PLCp as a plant community hydraulic trait allowed us to observe its interactions with more commonly used plant leaf traits and its direct effects on reduction in above-ground biomass production under drought. Our findings show that hydraulic traits are a promising tool to better understand the effects of drought at the species or community level (see also Brodribb 2017), and that mechanistic hydraulic trait-based modelling (Xu *et al*. 2016) could largely improve predictions of drought impacts on forage quality and quantity.

## Data

Data used in this manuscript were uploaded as **Supporting Information**.

## Sources of Funding

This study is part of the GrassAlt project funded by the Swiss National Science Foundation (SNF), grant n° CR31I3_156282/1. This study was also funded in part by the ‘Investments for the Future’ programme (grant no. ANR-10-EQPX-16, XYLOFOREST) from the French National Agency for Research and the Cluster of Excellence COTE (ANR-10-LABX-45, within the DEFI project) to S.D.

## Contributions by the Authors

A.V., C.D., C.S., A.R., M.M. and A.B.D. conceived the ideas and designed methodology; A.V., C.D., M.M., S.D., C.S. and L.L. collected the data; A.V. and P.M. analyzed the data and led the writing of the manuscript. All authors contributed critically to the drafts and gave final approval for publication.

## Conflict of Interest

None declared.

## Supplementary Material

Supplementary Dataset

Supplementary Material

## Supporting Information

The following additional information is available in the online version of this article—

**Table S1.** Summary table of the effects of seasonality, inter-annual variability and their interactions on plant species relative abundance, functional (SLA: surface leaf area, LDMC: leaf dry matter content) and hydraulic (PLCp: predicted percentage loss of conductivity) traits in control plots at Site 1 (Chéserex) and Site 2 (Saint-George).

**Table S2.** Summary of the experimental drought effects on soil moisture for the control (C), early- (Early) and late-season (Late) drought treatments along the season (Peak: peak of growing season, After peak: after peak of growing season and End: end of growing season) and the 2 years (2015 and 2016) at both sites (Site 1, Chéserex and Site 2, Saint-George).

**Figure S1.** Network of linkages between plant leaf economic (SLA, specific leaf area; LDMC, leaf dry matter content) and hydraulic (PLCp, predicted percentage loss of conductivity) traits and above-ground biomass under drought according to knowledge from the literature.

**Figure S2.** Relative abundance (±1 SE) of the most abundant species along the season (P: peak of growing season, AP: after peak of growing season and E: end of growing season) for the 2 years (2015 and 2016) at Site 1 (Chéserex) and the three drought treatments (Control, Early, Late).

**Figure S3.** Relative abundance (±1 SE) of the most abundant species along the season (P: peak of growing season, AP: after peak of growing season and E: end of growing season) for the 2 years (2015 and 2016) at Site 2 (Saint-George) and the three drought treatments (Control, Early, Late).
