# Supplementary material for: Responses of plant leaf economic and hydraulic traits mediate the effects of early- and late-season drought on grassland productivity
Source: AoB Plants. 2019 Apr 4;11(3):plz023. doi: 10.1093/aobpla/plz023 (PMC6499892; doi:10.1093/aobpla/plz023)

Vitra A, Deléglise C, Meisser M, Risch AC, Signarbieux C, Lamacque L, Delzon S, Buttler A, Mariotte P. (2019) Responses of plant leaf economic and hydraulic traits mediate the effects of early- and late season-drought on grassland productivity. *Annals of Botany Plants.*

Supplementary material

Table S1. Summary table of the effects of seasonality, inter-annual variability, and their interactions on plant species relative abundance, functional (SLA: surface leaf area, LDMC: leaf dry matter content) and hydraulic (PLCp: predicted percentage loss of conductivity) traits in control plots at Site 1 (Chéserex) and Site 2 (Saint-George). Seasonal (Season: Peak, After peak, End) and inter-annual (Year: 2015, 2016) variability effects were tested using linear mixed-effect model (packaged 'nlme') specifying 'block' as random factor. When necessary, data were log transformed to comply with the assumptions of normality and homoscedasticity. Statistical outputs are not shown for SLA of *Poa pratensis* and *Trifolium repens* at Site 1 since model residuals did not comply with the assumptions of normality. Overall, results show strong significant effects of the season on both plants species abundance and functional and hydraulic traits.

Site 1 - Chéserex

Species	Variability	PLCp		SLA		LDMC		Relative abundance	
Dactylis glomerata	Season	F2,9 = 4.197	P = 0.05	F2,18 = 17.775	**P < 0.001**	F2,18 = 8.232	**P < 0.01**	F2,18 = 34.822	**P < 0.001**
	Year	F1,9 = 6.038	**P = 0.04**	F1,18 = 5.309	**P = 0.03**	F1,18 = 0.009	P = 0.92	F1,18 = 5.845	**P = 0.03**
	Season x Year	F2,9 = 6.262	**P = 0.02**	F2,18 = 6.572	**P = < 0.01**	F2,18 = 5.332	**P = 0.02**	F2,18 = 2.176	P = 0.14
Lolium perenne	Season	F2,9 = 4.477	**P = 0.048**	F2,20 = 36.582	**P < 0.001**	F2,19 = 41.192	**P< 0.001**	F2,20 = 11.624	**P < 0.001**
	Year	F1,9 = 0.268	P = 0.62	F1,20 = 0.331	P = 0.57	F1,19 = 5.332	**P = 0.03**	F1,20 = 1.477	P = 0.24
	Season x Year	F2,9 = 1.755	P = 0.23	F2,20 = 2.195	P = 0.14	F2,19 = 4.219	**P = 0.03**	F2,20 = 1.270	P = 0.30
Phleum pratense	Season	F2,9 = 1.347	P = 0.31	F2,16 = 1.567	P = 0.24	F2,16 = 3.368	P = 0.06	F2,14 = 11.194	**P < 0.01**
	Year	F1,9 = 0.590	P = 0.46	F1,16 = 0.065	P = 0.80	F1,16 = 1.707	P = 0.21	F1,14 = 20.271	**P < 0.001**
	Season x Year	F2,9 = 2.797	P = 0.11	F2,16 = 1.241	P = 0.32	F2,16 = 3.105	P = 0.07	F2,14 = 0.847	P = 0.45
Poa pratensis	Season	F2,9 = 5.026	**P = 0.03**			F2,12 = 13.576	**P < 0.001**	F2,14 = 4.934	**P = 0.02**
	Year	F1,9 = 4.089	P = 0.07			F1,12 = 0.045	P = 0.84	F1,14 = 3.287	P = 0.09
	Season x Year	F2,9 = 2.912	P = 0.11			F2,12 = 2.994	P = 0.09	F2,14 = 2.487	P = 0.12
Taraxacum officinale	Season	F2,9 = 7.567	**P = 0.01**	F2,19 = 32.095	**P < 0.001**	F2,19 = 53.061	**P < 0.001**	F2,20 = 36.710	**P < 0.001**
	Year	F1,9 = 0.731	P = 0.42	F1,19 = 2.731	P = 0.12	F1,19 = 76.667	**P < 0.001**	F1,20 = 3.648	P = 0.07
	Season x Year	F2,9 = 1.820	P = 0.22	F2,19 = 15.758	**P < 0.001**	F2,19 = 27.222	**P < 0.001**	F2,20 = 1.406	P = 0.27
Trifolium repens	Season	F2,9 = 5.463	**P = 0.03**			F2,20 = 13.919	**P < 0.001**	F2,19 = 5.568	**P = 0.01**
	Year	F1,9 = 1.330	P = 0.28			F1,20 = 0.032	P = 0.86	F1,19 = 2.531	P = 0.13
	Season x Year	F2,9 = 0.336	P = 0.72			F2,20 = 1.134	P = 0.34	F2,19 = 1.186	P = 0.33

Site 2 - Saint-George

Species	Variability	PLC		SLA		LDMC		Relative abundance	
Dactylis glomerata	Season	F2,10 = 7.278	**P = 0.01**	F2,18 = 5.906	**P < 0.001**	F2,17 = 25.094	**P < 0.001**	F2,20 = 39.157	**P < 0.001**
	Year	F1,10 = 0.186	P = 0.68	F1,18 = 0.524	**P < 0.01**	F1,17 = 0.478	P = 0.50	F1,20 = 13.436	**P < 0.01**
	Season x Year	F2,10 = 12.370	**P < 0.01**	F2,18 = 2.040	P = 0.11	F2,17 = 4.974	P = 0.02	F2,20 = 6.090	**P < 0.01**
Lolium perenne	Season	F2,10 = 5.894	**P = 0.02**	F2,20 = 34.830	**P < 0.001**	F2,20 = 13.135	**P < 0.001**	F2,20 = 7.145	**P < 0.01**
	Year	F1,10 = 0.011	P = 0.92	F1,20 = 0.971	P = 0.34	F1,20 = 2.589	P = 0.12	F1,20 = 40.953	**P < 0.001**
	Season x Year	F2,10 = 1.053	P = 0.38	F2,20 = 7.308	**P < 0.01**	F2,20 = 16.749	**P < 0.001**	F2,20 = 1.618	P = 0.22
Poa pratensis	Season	F2,10 = 1.918	P = 0.20	F2,20 = 78.850	**P < 0.001**	F2,20 = 8.892	**P < 0.01**	F2,19 = 3.538	**P = 0.049**
	Year	F1,10 = 2.008	P = 0.19	F1,20 = 1.550	P = 0.23	F1,20 = 0.352	P = 0.56	F1,19 = 0.006	P = 0.94
	Season x Year	F2,10 = 2.019	P = 0.18	F2,20 = 1.568	P = 0.23	F2,20 = 2.738	P = 0.09	F2,19 = 1.269	P = 0.30
Trifolium repens	Season	F2,10 = 0.402	P = 0.68	F2,20 = 10.143	**P < 0.001**	F2,20 = 7.892	**P < 0.01**	F2,20 = 5.760	**P = 0.01**
	Year	F1,10 = 0.570	P = 0.47	F1,20 = 1.472	P = 0.24	F1,20 = 0.590	P = 0.45	F1,20 = 16.722	**P < 0.001**
	Season x Year	F2,10 = 0.960	P = 0.42	F2,20 = 0.277	P = 0.76	F2,20 = 3.395	P = 0.05	F2,20 = 13.751	**P < 0.001**

Table S2. Summary of the experimental drought effects on soil moisture for the control (C), early- (Early) and late-season (Late) drought treatments along the season (Peak: peak of growing season, After peak: after peak of growing season and End: end of growing season) and the two years (2015 and 2016) at both sites (Site 1, Chéserex and Site 2, Saint-George). The mean soil moisture (± 1SE) has been performed by TDR measurements. The difference between control and drought is the mean percentage difference of soil moisture (± 1SE) between drought plots and their respective control plots (note that only the current drought of each time period appears, *i.e.* Early at Peak and Late in After Peak). The difference between control and drought after two months is the mean percentage difference of soil moisture (± 1SE) between drought plots two months after the end of the drought (*i.e.,* during this two months drought plots are watered similarly to control plots) and their respective control plots (note that only the two months after drought recovery treatment of the corresponding time period appears, *i.e.,* Early at After peak and Late at End).

(Table S2)

Site	Year	Season	Timing of drought	Mean soil moisture (Volumetric water content in %) ± Standard error	Difference between Control and Drought ± Standard error	Difference between Control and Drought after two months ± Standard error
Site 1 - Chéserex	2015	Peak	**Early**	**17.0 ±1.3**	***-50 %* ±2**	
			Late	27.4 ±1.8		
			Control	33.8 ±2.6		
		After peak	Early	11.7 ±0.8		*-0 %* ±13
			**Late**	**4.9 ±0.4**	***-57 %* ±9**	
			Control	12.0 ±1.4		
		End	Early	14.5 ±0.8		
			Late	14.3 ±0.7		*-14 %* ±3
			Control	16.8 ±1.5		
	2016	Peak	**Early**	**11.2 ±1.1**	***-55 %* ±5**	
			Late	22.8 ±4.1		
			Control	24.9 ±1.3		
		After peak	Early	11.7 ±1.8		*-3 %* ±5
			**Late**	**7.7 ± 0.2**	***-34 %* ±7**	
			Control	12.0 ±1.3		
		End	Early	14.9 ±2.2		
			Late	12.0 ±0.7		*-16 %* ±12
			Control	14.7 ±1.5		
Site 2 – Saint-George	2015	Peak	**Early**	**17.4 ±0.9**	***-63 %* ±1**	
			Late	39.5 ±2.1		
			Control	47.1 ±4.2		
		After peak	Early	18.1 ±0.6		*-8 %* ±2
			**Late**	**10.5 ±1.1**	***-46 %* ±7**	
			Control	19.7 ±0.7		
		End	Early	29.6 ±2.0		
			Late	23.1 ±1.2		*-24 %* ±5
			Control	30.5 ±1.5		
	2016	Peak	**Early**	**15.4 ±1.2**	***-48 %* ±3**	
			Late	25.2 ±3.1		
			Control	29.9 ±1.1		
		After peak	Early	25.4 ±2.8		*-2 %* ±1
			**Late**	**11.3 ±2.3**	***-56 %* ±6**	
			Control	26.0 ±3.1		
		End	Early	32.5 ±1.7		
			Late	20.7 ±1.6		*-38 %* ±7
			Control	33.5 ±3.6		

Figure S1. Network of linkages between plant leaf economic (SLA, specific leaf area; LDMC, leaf dry matter content) and hydraulic (PLCp, predicted percentage loss of conductivity) traits and aboveground biomass under drought according to knowledge from the literature. Signs indicate positive (+) or negative (-) correlations between variables and numbers refer to the literature supporting the expected links between variables.

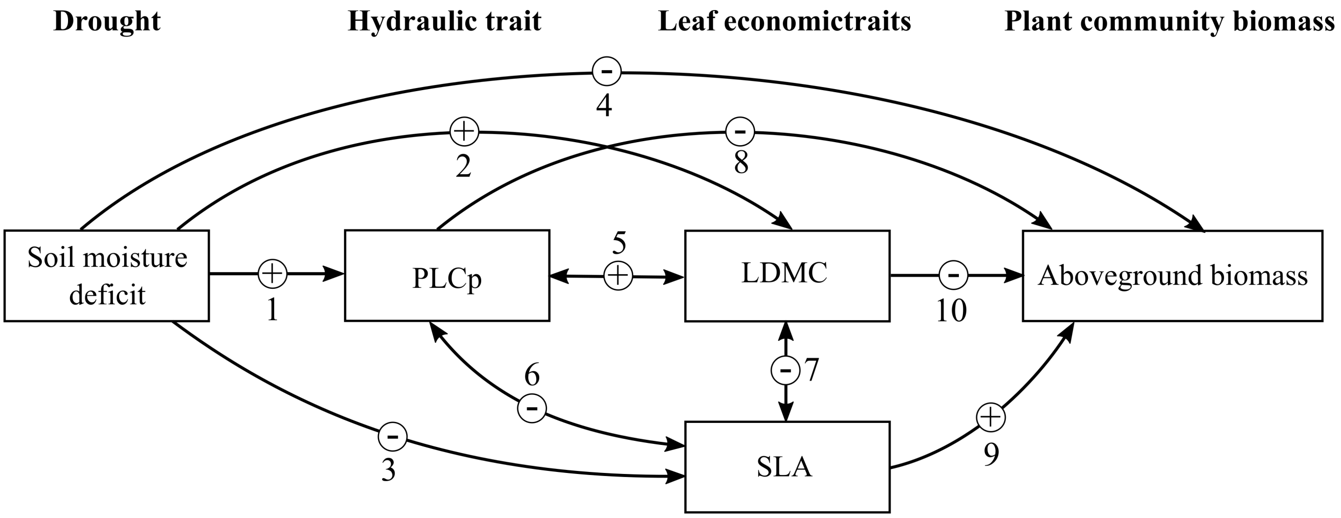

1 – Anderegg et al., 2016; Choat et al., 2012; Cochard et al., 1996.

2 – Deléglise et al., 2015; Jung et al., 2014.

3 – Cornwell and Ackerly 2009; Deléglise et al., 2015; Griffin-Nolan et al, 2018; Jung et al., 2014.

4 – Bloor et al., 2010; Buttler et al., 2019; Gilgen and Buchmann, 2009; Kahmen et al., 2005; Mariotte et al., 2013.

5 – Kursar et al., 2009; Vinya et al., 2012.

6 – Kursar et al., 2009; Pérez-Ramos et al., 2013; Villagra et al., 2013; Vinya et al., 2012.

7 – Deléglise et al., 2015; Jung et al., 2014; Pontes et al., 2007.

8 – Chaves et al., 2003; Choat et al., 2012.

9 – Deléglise et al., 2015; Wellstein et al. 2017.

10 – Deléglise et al., 2015.

References are indicated in full in the manuscript.

Figure S2. Relative abundance (± 1SE) of the most abundant species along the season (P: peak of growing season, AP: after peak of growing season and E: end of growing season) for the two years (2015 and 2016) at Site 1 (Chéserex) and the three drought treatment (Control, Early, Late). For each sampling time, drought treatment effect has been tested using ANOVA specifying 'Drought treatment' as fixed factor and 'Block' as error term. When drought treatment had significant effects on species abundance, significant differences between the three drought treatments have been evaluated by Tukey test. For each sampling time, points with different letter are significantly different ($P < 0.05$). Overall, drought (early- or late-season) did not significantly impact species relative abundance by comparison to the control (except in late-season drought for *Lolium perenne* in 2015 and *Taraxacum officinale* in 2016).

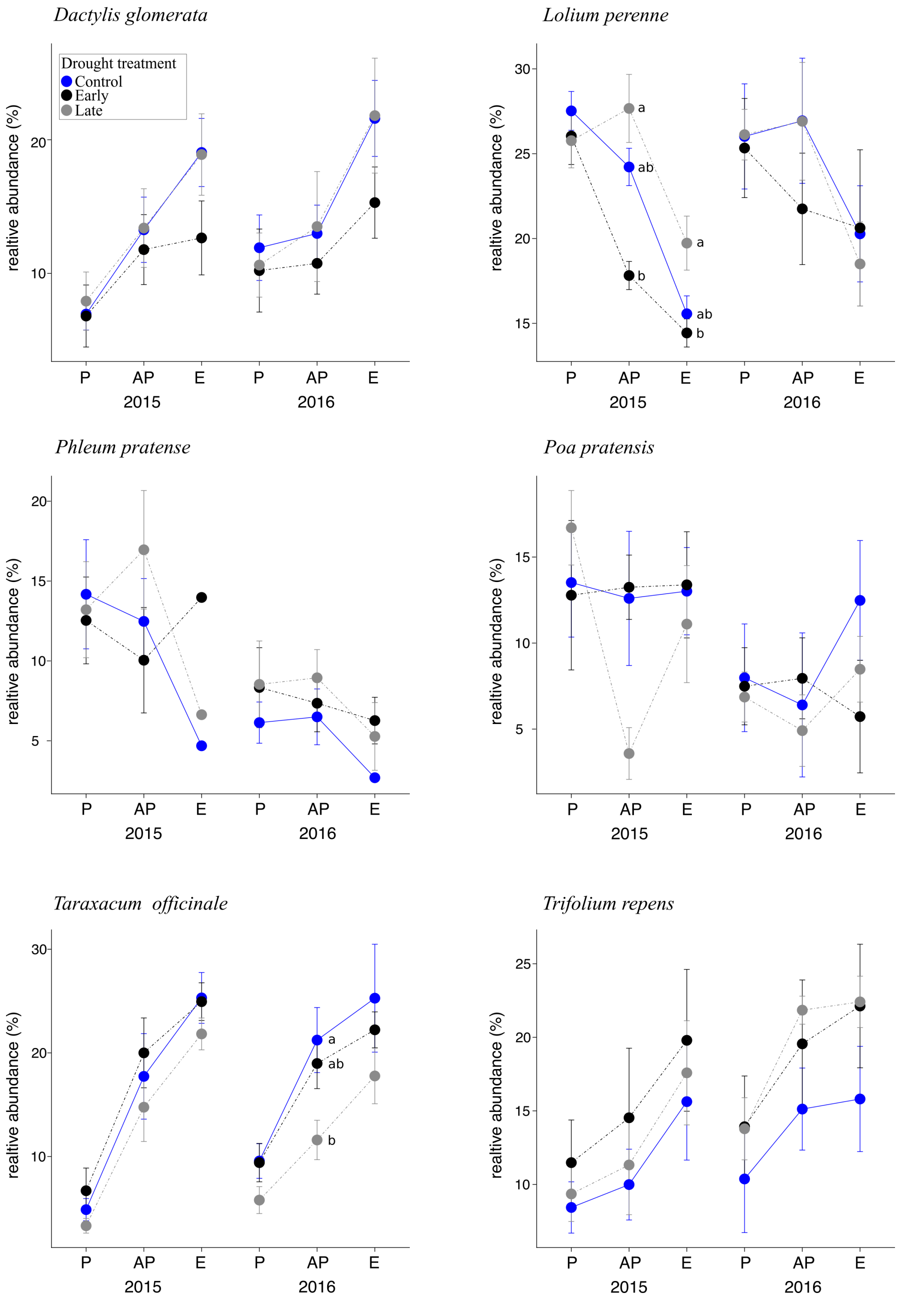

Figure S3. Relative abundance (± 1SE) of the most abundant species along the season (P: peak of growing season, AP: after peak of growing season and E: end of growing season) for the two years (2015 and 2016) at Site 2 (Saint-George) and the three drought treatments (Control, Early, Late). For each sampling time, drought treatment effect has been tested using ANOVA specifying 'Drought treatment' as fixed factor and 'Block' as error term. When drought treatment had significant effects on species abundance, significant differences between the three drought treatments have been evaluated by Tukey test. For each sampling time, points with different letter are significantly different (P < 0.05). Overall, drought (early- or late-season) did not significantly impact species relative abundance by comparison to the control (except at the end of the season after a late-season drought for *Trifolium repens* in 2015 and *Poa pratensis* in 2016).

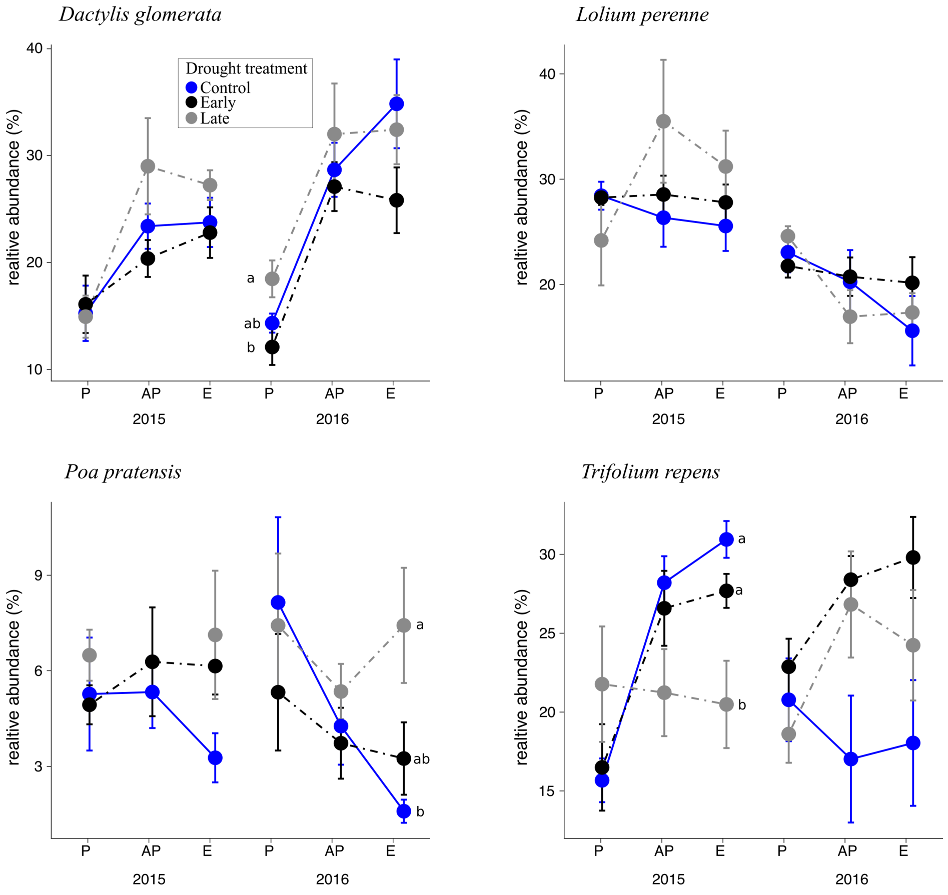

Supplement: Supplementary Material [file plz023_suppl_supplementary_material.pdf]
